# Threshold Cascade Dynamics in Coevolving Networks

**DOI:** 10.3390/e25060929

**Published:** 2023-06-13

**Authors:** Byungjoon Min, Maxi San Miguel

**Affiliations:** 1 Department of Physics, Chungbuk National University, Cheongju 28644, Chungbuk, Republic of Korea; 2 Research Institute for Nanoscale Science and Technology, Chungbuk National University, Cheongju 28644, Chungbuk, Republic of Korea; 3 IFISC (CSIC-UIB), Institute for Cross-Disciplinary Physics and Complex Systems, Campus Universitat Illes Balears, E-07122 Palma, Spain

**Keywords:** coevolution, threshold cascades, link rewiring

## Abstract

We study the coevolutionary dynamics of network topology and social complex contagion using a threshold cascade model. Our coevolving threshold model incorporates two mechanisms: the threshold mechanism for the spreading of a minority state such as a new opinion, idea, or innovation and the network plasticity, implemented as the rewiring of links to cut the connections between nodes in different states. Using numerical simulations and a mean-field theoretical analysis, we demonstrate that the coevolutionary dynamics can significantly affect the cascade dynamics. The domain of parameters, i.e., the threshold and mean degree, for which global cascades occur shrinks with an increasing network plasticity, indicating that the rewiring process suppresses the onset of global cascades. We also found that during evolution, non-adopting nodes form denser connections, resulting in a wider degree distribution and a non-monotonous dependence of cascades sizes on plasticity.

## 1. Introduction

Understanding collective interactions among agents is crucial for predicting the behavior of complex systems [1,2,3,4,5]. Recently, studies of group and higher-order interactions have received significant interest in the study of the statistical physics of complex systems [5,6,7,8]. Social contagion is one of the most interesting examples of group interactions, underlying the spread of information, fads, opinions, or behaviors [1,2,3,9,10,11,12,13,14]. Unlike the simple contagion process for the spread of infectious diseases which occurs via pairwise interactions [15], social complex contagion [3,4,6] usually requires simultaneous interactions with multiple neighbors. The threshold model is a pioneering work in the field of complex contagion describing cascading dynamics [1,2,9,10]. It is a binary-state model in which the adoption of an initial minority state by a node in an interaction network requires that the fraction of neighboring nodes that have already adopted that state exceeds a threshold value. Cascade phenomena described by this model can represent not only the spread of social behaviors but also the transmission of neural signals [16], error propagation in financial markets [17], and the collapse of power grids [18].

Although many studies have been conducted on threshold cascade models [2,10,19,20,21,22,23], including competition of simple and complex contagion processes [24,25,26], most have focused only on the dynamics on static networks [2,10,19,20,21,22,23]. However, real-world complex systems change their connection patterns and the network of interactions changes dynamically [27,28,29,30]. In this respect, some studies have attempted to analyze coevolutionary dynamics, that is, dynamical processes in which the time evolution of the states of the nodes and the evolution of the network topology are dynamically coupled. These include coevolving voter models [31,32,33,34,35,36,37,38], coevolving spin systems [39,40], coevolving models of opinion formation [41,42], epidemic models of adaptive networks [43,44,45], coevolving models of cultural evolution [46,47], and game theoretical models [48]. While we here focus on the coevolution of node states and network topology, there have been studies that address the coevolution between different dynamical processes in a static network [49,50,51,52]. In cases where cascading dynamics are coupled with the evolution of the network structure, it is essential to understand the coevolutionary dynamics of the network topology and threshold dynamics. However, only a few studies have been conducted on models of coevolutionary dynamics including group or collective interactions [40,53]. Here, we attempt to understand the behavior of the threshold cascade model by incorporating the adaptive dynamics of the network topology. This is a tool for a better understanding of the comparison of threshold models with empirical data [3,11,54,55,56,57,58,59,60].

In this study, we propose a coevolving threshold cascade model, where the nodes are in two possible states and can redefine their connections in the network depending on the dynamical states of the nodes. Initially, only a few seed nodes in a network are in a minority state that can represent new information, opinions, or innovations that might spread into the system. According to the threshold process, given a node *i* in the initial majority state, if the fraction of its neighbors that are already in the new initial minority state exceeds a certain threshold θ, the node *i* changes state, and becomes “adopting”. In addition, by following the homophilic tendencies observed in society [61,62], an agent may reduce its social ties with individuals who are in an opposite state and establish new connections at random with agents who share the same state. To be specific, when a node *i* is adopting, then a non-adopting node from the neighbors of node *i* breaks its link with node *i* and establishes a new link with a non-adopting node in the network. Therefore, the evolution of the network topology by link rewiring is coupled with the complex contagion processes so that the network structure constantly evolves in response to changes in the behavior of its constituents. The main result obtained from simulations of this model, which is well described by an appropriate mean-field theoretical approach, is that the rewiring process can suppress the emergence of global cascades by a mechanism of the segregation of adopting nodes.

## 2. Model

**Figure 1:**
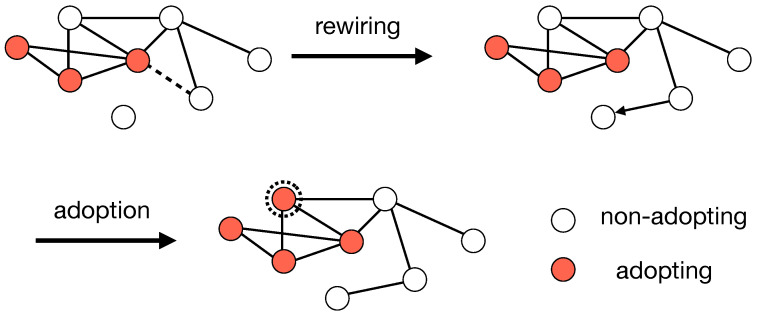
An example of the evolution rules of the coevolutionary dynamics of a threshold cascade model. A connected pair of an adopting (filled circles) and a non-adopting node (open circles) is removed with a probability *p*, and the non-adopting node establishes a connection to a new node that is not adopting, chosen randomly from the entire network. In addition, a non-adopting node becomes adopting if the fraction of adopting neighbors is larger than the threshold θ. Once a node becomes adopting, the node is then permanently in this state.

We consider a coevolving threshold cascade model as shown in Figure 1. The coevolutionary threshold model consists of two parts: (i) the rewiring of links and (ii) the adoption of a new state (opinion, idea, or innovation). Dynamics start from seed node initiators: a small fraction $R_{0}$ of randomly selected adopting nodes. Furthermore, the dynamics proceed by the specific rules below.

Link rewiring adaptively changes the structure of the network representing the situation in which an agent meets a new possible state, but does not want to adopt it. At each time step of a random sequential update, each link that connects a pair of an adopting and a non-adopting node is removed with probability *p*. In addition, the non-adopting node that loses a link establishes a new link with a node that is not currently adopting, chosen randomly from the entire network. The parameter *p*, called the network plasticity, is a measure of the ratio of the timescales of network evolution to the adoption dynamics.

The adoption of a new state is a complex contagion process following the dynamics of Granovetter’s and Watts’ threshold model [1,2], where a non-adopting node becomes adopting if the fraction of its adopting neighbors exceeds a threshold θ. We assume that each node has the same threshold θ. Once nodes are adopting, their adopting state remains permanently. The two processes of link rewiring and adoption proceed until there are no active links connecting a pair of adopting and non-adopting nodes in a network.

## 3. Results

### 3.1. On a Static Network

To establish a benchmark for comparison, we begin by analyzing the threshold dynamics on a static (non-adaptive) network. This is a well-established model to explain the onset of the extensive size of the cascade of adoption from a few seed nodes, referred to as a “global cascade” [2,10]. Typically, the global cascade occurs in a specific domain of two parameters: network connectivity and threshold. For instance, in Erdös–Rényi (ER) graphs, when the average degree *z* is less than the percolation threshold $z_{1}$ of the graphs, global cascades do not occur, as there is no giant connected component. In addition, when *z* is greater than a second threshold $z_{2}$ which depends on the threshold θ, the nodes that exceed their threshold are rare because the network becomes too dense. Therefore, global cascades can occur only in the range between $z_{1}$ and $z_{2}$.

For local tree-like networks, the transition lines in the parameter space between the global cascade and no cascade domains can be precisely identified using a mean-field analysis [2,10]. On a random graph, the average fraction of the adopting nodes in a stationary state, called the cascade size *R*, is given by the probability of a randomly selected node to become adopting. The size *R* can be obtained by approximating the network as a tree, with a chosen node as the root and considering the cascade of adoption towards the root. For a fixed degree distribution P(k) and initial seed fraction $R_{0}$, such a probability is given by [10]:

(1) R=R0+(1−R0)∑k=0∞P(k)∑m=0kkmq∞m(1−q∞)k−mF(m/k,θ),

where $q_{\infty }$ represents the probability that a node, reached via a randomly selected link, is adopting in the stationary state and F(m/k,θ) is the threshold function. To be specific, if m/k>θ, F(m/k,θ)=1, otherwise F(m/k,θ)=0.

The probability $q_{\infty }$ is computed by solving the following self-consistency equation iteratively [10],

(2) qn=q0+(1−q0)∑k=0∞kP(k)z∑m=0k−1k−1mqn−1m(1−qn−1)k−m−1F(m/k,θ),

where $q_{n}$ is the probability of step *n* and $q_{0}=R_{0}$. In the limit n→∞, we can obtain the probability $q_{\infty }$ in the steady state. In addition, mean-field theory predicts the necessary conditions for global cascades from the linear stability analysis of a trivial fixed point $q_{\infty }=0$ in the limit $R_{0}\to 0$ as:

(3) $$\begin{array}{c} \sum_{k=1}^{\infty }\frac{k(k-1)}{z}P\left(k\right)F\left(1/k,\theta \right)>1. \end{array}$$

Using this transition point and the size of adopting nodes predicted from the above theory as benchmarks, we will now analyze how they are modified by the coevolutionary adaptive dynamics of the network.

### 3.2. Segregation of Adopting Nodes via Link Rewiring

**Figure 2:**
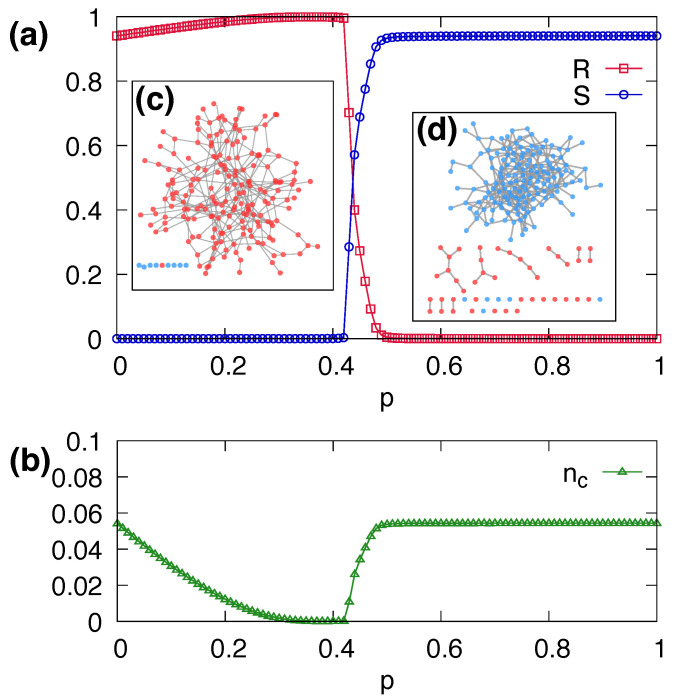
(**a**) The final fraction *R* of adopting nodes and the size *S* of the largest non-adopting cluster as a function of the network plasticity *p*. (**b**) The number $n_{c}$ of clusters to network size *N* as a function of *p*. The dynamics starts with θ=0.18 in ER networks with $N=10^{5}$, z=3, and an initial fraction of seeds of $R_{0}=2\times 10^{-4}$. The average values are obtained by $10^{4}$ independent runs with different network realizations for each run. Examples of network structures with N=200 at the steady state with (**c**) p=0.2 and (**d**) p=0.8. Red and blue nodes represent adopting and non-adopting states, respectively.

We have explored the coevolutionary threshold dynamics with link rewiring in Erdös–Rényi (ER) networks with $N=10^{5}$, z=3, and θ=0.18. We set the initial fraction of seeds as $R_{0}=2\times 10^{-4}$. To start with, we measured the global cascade size *R* as a function of network plasticity *p* in order to examine the effect of link rewiring. We also computed the size *S* of the largest cluster composed of non-adopting nodes in order to inspect the network structure. The size of adopting nodes *R* and the largest non-adopting cluster *S* in a steady state is shown in Figure 2a as a function of *p*. Note that the case of p=0 corresponds to the result of threshold cascading dynamics in a static network.

We found a transition between a global cascade and no cascade for a critical value $p_{c}$ of the plasticity. When $p<p_{c}$, most nodes are adopting, forming a large connected cluster of adopting nodes. Almost all nodes belong to a single cluster when p≈0.4. As *p* further increases, adopting nodes are separated from the large cluster due to rewiring. Beyond the transition point, the cascading dynamics originating from the seed nodes fail to propagate throughout the entire network. As a result, many small adopting clusters appear and a large cluster composed of non-adopting nodes emerges.

Figure 2b shows the number of clusters $n_{c}$ normalized to the total number of nodes *N* in a steady state as a function of network plasticity *p*. For small values of *p*, $n_{c}$ decreases as *p* increases. That is, small non-adopting clusters gradually join adopting clusters as *p* increases due to rewiring. Around p≈0.4, almost all nodes belong to a single adopting cluster, and therefore $n_{c}\approx 0$. As *p* increases beyond the transition point, adopting nodes become segregated due to rewiring and small adopting clusters appear. Examples of network structures at a steady state are shown in Figure 2c for p=0.2 and Figure 2d for p=0.8. When p=0.2 in Figure 2c, there exists a single large cluster of adopting nodes. On the other hand, when p=0.8 in Figure 2d, adopting nodes are segregated, resulting in a low *R* value. Therefore, we find that in the “global cascade” phase, there is one large adopting cluster, whereas in the “no cascade” phase there is a large non-adopting cluster and many small adopting clusters. In summary, a mechanism for the transition to the “no cascade” phase in the coevolutionary model is the segregation of adopting nodes via link rewiring.

### 3.3. Phase Diagram for Global Cascades

**Figure 3:**
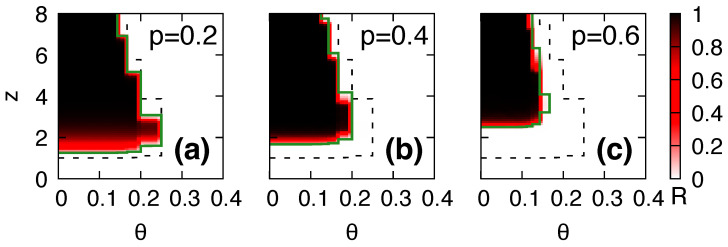
The final fraction *R* of adopting nodes in ER networks with $N=10^{5}$ as a function of the average degree *z* and threshold θ, with various rewiring probabilities, i.e., (**a**) p=0.2, (**b**) p=0.4, and (**c**) p=0.6, in a steady state. Th dashed lines represent the transition points between the global cascade and no cascade phases in static networks, that is p=0, obtained from Equation (3). The solid lines represent the transition points with network plasticity *p* by using mean-field approximations. The numerical results are obtained by $10^{3}$ independent runs with different network realizations for each run.

We conducted numerical simulations to determine the fraction *R* of the adopting nodes in the steady state by varying the average degree *z*, the adoption threshold θ, and rewiring probabilities p=0.2,0.4, and 0.6 using ER graphs with $N=10^{5}$ and $R_{0}=2\times 10^{-4}$ (Figure 3). The dashed lines in Figure 3 represent the location of the transition lines between the “global cascade” and “no cascade” phases in static networks as obtained from Equation (3). One of the key findings is that the domain of global cascades shrinks with increasing network plasticity *p*. Specifically, for a fixed threshold θ, as *p* increases, the first transition point $z_{1}$ of the mean degree increases, whereas the second transition point $z_{2}$ decreases. The first threshold $z_{1}$ for the global cascades becomes delayed with increasing *p* because the rewiring of links effectively segregates the adopting nodes, as described in the previous section. In addition, the second threshold $z_{2}$ decreases because the nodes that exceed their threshold also become rare due to link rewiring *p*. Unlike coevolving simple contagion models [29], the second transition $z_{2}$ is a peculiar feature of threshold models.

Our finding shows that link rewiring suppresses the emergence of global cascades as compared to what occurs in a static network. This is because the rewiring process removes the links that connect adopting and non-adopting nodes. Consequently, the cascading dynamics become segregated and cannot propagate further. Therefore, the adaptive mechanism enabled by the rewiring process effectively suppresses global cascades by removing active links, i.e., links that connect adopting and non-adopting nodes. This mechanism allows the network to reorganize itself in response to the changes in the state of the nodes, effectively preventing the spread of a new state.

### 3.4. Non-Monotonicity in the Size of the Global Cascade

**Figure 4:**
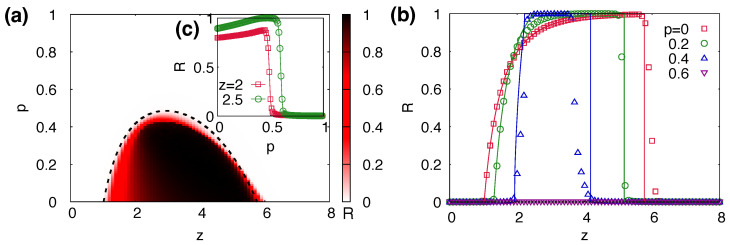
(**a**) The final size *R* of cascades as a function of network plasticity *p* and average degree *z* of the ER networks with threshold θ=0.18 and seed fraction $R_{0}=2\times 10^{-4}$. The dashed lines represent analytical predictions obtained by Equation (9). (**b**) The size *R* as a function of the average degree *z* in ER networks for p=0,0.2,0.4, and 0.6 with θ=0.18. The lines represent analytical predictions based on Equations (4) and (5). (**c**) Inset shows the size *R* with respect to the probability of link rewiring *p* for z=2 and 2.5 and θ=0.1. The numerical results were obtained with $10^{3}$ independent runs with different network realizations for each run.

While the area of the parameter space (*z*,θ) in which global cascades occur decreases monotonically with the increasing network plasticity *p*, the size *R* of the global cascades exhibits more complex patterns. One could expect that the size *R* also decreases monotonically with increasing *p*, but we found that *R* can increase with increasing *p* within a certain range of *p* in the global cascade phase. Figure 4a shows the size *R* of cascades as a function of *p* and *z*, with $R_{0}=2\times 10^{-4}$, $N=10^{5}$, and θ=0.18 in ER networks. As *p* increases, the value of $z_{1}$ at which the global cascades begin to occur is delayed. However, when the global cascade is initiated, the rate of increase in *R* is greater for larger values of *p*, as shown in Figure 4b. Hence, in the region in which 2≲z≲4, we show that the cascade size *R* increases as the link rewiring probability *p* increases. Figure 4c shows *R* as a function of *p* with θ=0.1 and z=2 and 2.5 in the region where the increase in *R* with *p* is maximized. In this figure, *R* increases as the plasticity *p* increases below the transition point to the “no cascade” phase.

The increase in *R* with increasing *p* can occur when separated non-adopting clusters are connected to the giant cluster through new connections established during link rewiring. This effect can be characterized by considering the number $n_{c}$ of clusters, as shown in Figure 2b. The fraction $n_{c}$ of the clusters decreases as *p* increases from p=0. This implies that increasingly more small clusters merge into the giant connected component of the network as *p* increases, thereby promoting the larger size of cascading dynamics. For instance, when p=0.4, the separated nodes cease to exist, indicating that initially separated nodes have become linked to a cluster via link rewiring.

### 3.5. Structure of Rewired Networks

**Figure 5:**
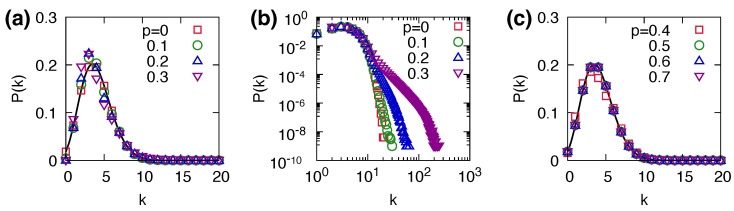
Degree distribution P(k) of the coevolving threshold model at the steady state in (**a**) linear and (**b**) log scales for p=0,0.1,0.2,0.3 (global cascade region) and (**c**) linear scale for p=0.4,0.5,0.6,0.7 (no cascades region). The results were obtained from ER networks with z=4 and $N=10^{5}$ with $10^{4}$ independent runs. The solid lines in (**a**,**c**) represent the Poisson distribution with z=4.

We examined the network structure in the steady state. We found that the degree distribution broadens as *p* increases. Figure 5 shows the degree distribution P(k) in the steady state for various values of *p*, where the dynamics start from ER networks with z=4 and $N=10^{5}$. As *p* increases, the distribution deviates from a Poisson distribution as indicated by the solid line in Figure 5a. In particular, the probability of finding a large degree value *k* increases with *p*, which leads to a broader degree distribution. This implies that as the network plasticity *p* increases, the degree distribution becomes broader owing to rewiring.

In our coevolving threshold model, non-adopting nodes remove their links to adopting nodes and then randomly connect to a new non-adopting neighbor. Through this process, the link density of the non-adopting nodes continuously increases over time. Consequently, nodes with higher degrees gradually appear during the evolution. Furthermore, this continuous increase in link density and the emergence of higher degree nodes intensify the overall connectivity of non-adopting nodes, potentially promoting the larger size of cascading dynamics. On the other hand, in the no cascades phase, there are no adopting nodes of extensive size; therefore, the number of active links that can be potentially rewired is limited. Therefore, the degree distribution in this region remains approximately a Poisson distribution (Figure 5c).

### 3.6. Mean-Field Approximations

Finally, we propose a mean-field approximation of the coevolving threshold model on random networks that accounts for our numerical results. We consider the effects of link rewiring by generalizing the mean-field equations for the static networks. In our model, there are two main effects of link rewiring: removing active links between adopting and non-adopting nodes and increasing the density of links between non-adopting nodes as new links are created. By implementing these two effects, we modify the self-consistency equation (Equation (2)) for the probability $q_{n}$ in a local tree-like network as

(4) qn=(1−p˜)q0+(1−p˜)(1−q0)∑k=0∞kQ(k,n)z∑m=0k−1k−1mqn−1m(1−qn−1)k−m−1F(m/k,θ),

where Q(k,n) is the degree distribution of the non-adopting nodes at time step *n* and $\tilde{p}$ is the probability that an active link will be removed before a non-adopting node at one end of the link is adopting. Note that unlike the threshold model in a static network, the degree distribution is neither time independent nor initially given because of the rewiring processes. Similarly, the mean-field equation of the cascade size at step *n* is approximately given by

(5) Rn=R0+(1−R0)∑kQ(k,n)∑m=0kkmqnm(1−qn)k−mF(m/k,θ),

where $R_{n}$ is the fraction of adopting nodes at time step *n*.

We suggest a zero-th order estimation of the probability of link removal $\tilde{p}$ and the time-dependent degree distribution Q(k,n). If an adopting node is connected to a non-adopting node, the link between them is removed with probability *p* at each time step. Therefore, in order to make an accurate prediction of $\tilde{p}$, it is necessary to know the time interval required for a non-adopting node to become adopting. However, this interval is difficult to predict, because the value is determined by collective interactions and not by the properties of individual links. To qualitatively explore the effect of link rewiring, we assume that link rewiring only affects one time step, leading to $\tilde{p}\approx p$. This assumption underestimates the actual value of $\tilde{p}$ because the active links can persist for multiple steps. However, it can qualitatively explain the effect of active link removal as an approximation.

Subsequently, we estimated the time-dependent degree distribution Q(k,n) by adding a new connection randomly among non-adopting nodes. As the time step *n* increases, the average degree of non-adopting nodes also increases. In well-mixed populations consisting of *N* nodes, the degree distribution of non-adopting nodes at step *n* can be approximated as follows:

(6) Q(k,n)=Nnkπnk(1−πn)Nn−k,

where $N_{n}$ is the number of non-adopting nodes ($N_{n}=\left(1-R_{n}\right)N$) and $\pi_{n}$ is the probability that two randomly chosen non-adopting nodes are connected at time *n*. To estimate the probability $\pi_{n}$, we assume again that the effect of link rewiring lasts for only one time step. Thus, the probability $\pi_{n}$ can be approximated as follows:

(7) $$\begin{array}{c} \pi_{n}=\pi_{n-1}\left[1+\frac{p\left(1-\theta \right)\Delta R_{n-1}}{1-R_{n-1}}\right], \end{array}$$

where $\Delta R_{n-1}$ is the change in *R* between the steps n−1 and *n*. The estimation is based on the assumption that the number of additional links between the non-adopting nodes is equal to the number of links lost by the adopting nodes during link rewiring. The term (1−θ) represents the maximum fraction of active links in an adopting node that are subject to link rewiring, because at least θ fraction of links are already connected to adopting neighbors.

Combining Equations (6) and (7), we can estimate the final fraction $R_{\infty }$ of the adopting nodes in the steady state by iteratively solving Equations (4) and (5). A comparison of the theoretical predictions for *R* and the numerical simulation results is shown in Figure 4b. The lines in Figure 4b corresponding to the analytical results give a good description of the main features of the numerical results. Our approximation accounts for the two dynamical effects of link rewiring: one segregates the adopting nodes by removing the active links, and the other increases the link density of non-adopting nodes, which could promote contagion above the first transition $z_{1}$. The quantitative discrepancies between our mean-field approximation and numerical results are primarily caused by the assumptions that we made to derive $\tilde{p}$, Q(k,n) and the term (1−θ) in $\pi_{n}$.

In addition, the necessary condition for global cascades in the limit $R_{0}\to 0$ can be predicted by a linear stability analysis of a trivial fixed point $q_{\infty }=0$ as follows:

(8) $$\begin{array}{c} \left(1-\tilde{p}\right)\sum_{k=1}^{\infty }\frac{k(k-1)}{z}P\left(k\right)F\left(1/k,\theta \right)>1. \end{array}$$

In order for a global cascade to occur, an initial cascade must be triggered; hence, we estimate the necessary condition using the degree distribution with n=0, P(k). The cascading condition in the coevolutionary dynamics is approximately modified by a factor of $\left(1-\tilde{p}\right)$ from the condition in static networks. This implies that the cascading condition of the coevolutionary cascading model obtained with the mean-field approximation predicts the condition for the cascading dynamics that involves random link removals with a probability $\tilde{p}$. When we approximate $\tilde{p}\approx p$, the transition point can be estimated. The transition points between global cascade and no cascade phases predicted by the theory are denoted by lines in Figure 3 and Figure 4a. Overall, our mean-field approximations give reasonable predictions. We can predict the critical value of network plasticity, denoted as $p_{c}$, as:

(9) $$\begin{array}{c} p_{c}=1-{\left[\sum_{k=1}^{\infty }\frac{k(k-1)}{z}P\left(k\right)F\left(1/k,\theta \right)\right]}^{-1}. \end{array}$$

**Figure 6:**
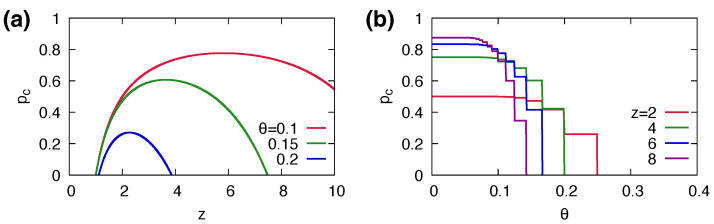
The critical values $p_{c}$ of network plasticity estimated by the mean-field approximation (**a**) with respect to *z* with fixed θ=0.1,0.15,0.2 and (**b**) with respect to θ with fixed z=2,4,6,8.

The critical values $p_{c}$ with respect to *z* with fixed θ and with respect to θ with fixed *z* are shown in Figure 6.

## 4. Summary and Discussion

We have studied the coevolutionary dynamics of network topology and social complex contagion using a binary-state threshold cascade model. We investigated how the mechanism for a global cascade is modified by the dynamics of the network topology and also the asymptotic stationary state of the network structure. Network dynamics, characterized by a plasticity parameter *p*, follow from a rewiring of links to cut the connections between nodes in different states. We find that the network dynamics suppress the onset of global cascades; there is a transition from a “global cascade” state to a “no cascade” state as the network plasticity *p* is increased beyond a critical value $p_{c}$, so that the domain of parameters (threshold θ and network mean degree *z*) for which global cascades occur shrinks compared to the situation in a static network. We have found that non-adopting nodes become more densely connected during evolution, leading to a broader degree distribution and to a non-monotonous dependence of cascades sizes on plasticity *p* within the “global cascade” phase. We have also developed a mean-field approximation that provides a good description of the transition lines between the “global cascade” and “no cascade” phases in the presence of link rewiring.

In previous models of coevolving voter dynamics, a generic result, different to what we find here, was the existence of a network fragmentation transition in two main network components [32,36]. However, these studies considered binary-state models with two equivalent states, while here we consider the spreading of an initial minority state with threshold dynamics in which a change of state is only allowed from the initial majority state to the minority state. Additionally, unlike coevolving epidemic models with simple contagion [29], once a node becomes adopting, it remains in that state permanently in our model. The consequence is that there is always a large network component and small segregated clusters, some of which are reminiscent of the shattered fragmentation transitions found in multilayer coevolution [34,38]. Overall, this study offers insights into the coevolutionary dynamics of social complex contagion and network evolution for an understanding of complex and evolving systems. In addition, this study provides a framework for studying and controlling the cascading phenomena in real-world systems, highlighting the importance of the interplay between network dynamics and social complex contagion.

## Data Availability

Data is contained within the article.
